# Supplementary material for: Efficient noise mitigation technique for quantum computing
Source: Sci Rep. 2023 Mar 8;13:3912. doi: 10.1038/s41598-023-30510-5 (PMC9995348; doi:10.1038/s41598-023-30510-5)
Supplement: Supplementary file 1 — Supplementary Information 1. [file 41598_2023_30510_MOESM1_ESM.pdf]

Supplementary Information for Efficient Noise Mitigation Technique for Quantum Computing

Ali Shaib, Mohamad Hussein Naim, Mohammed E. Fouda, Rouwaida Kanj, and Fadi Kurdahi

This supplementary document supports the discussion in the main text by providing theoretical proof and technical details. Section 1 provides the proof of Lemma 1. Section 2 provides information about the Jensen-Shannon Divergence (JSD). Section 3 summarizes our proposed algorithm for the construction of Q_m. Section 4 compares the performance of Q_m between using $p(|\boldsymbol{in}\rangle)$ and p_{ave}. Section 5 presents the average JSD and standard deviation for each input state $|\boldsymbol{in}\rangle$ over different depths.

I. Proof of Lemma 1

Lemma 1: Let $\boldsymbol{\lambda}$ and $\boldsymbol{p}$ be the respective eigenvalues and error rates of a Pauli Channel with n qubits, then

$$\boldsymbol{\lambda}^m = \boldsymbol{W}\boldsymbol{M}^m |0\rangle$$

where $\boldsymbol{W}$ is a Walsh-Hadamard Transform, and $\boldsymbol{M}$ is a $2^n \times 2^n$ matrix such that $M_{ij} = p_{i \oplus j}$ ($\oplus$ is the bitwise exclusive-OR operator).

Proof of Lemma 1: For the LHS of Lemma 1, we have

$$\boldsymbol{\lambda} = \boldsymbol{W}\boldsymbol{p} \tag{1}$$

Then

$$\lambda_i = \sum_{k=0}^{2^n-1} (-1)^{ik} p_k \tag{2}$$

And,

$$\lambda_i^m = \left(\sum_{k=0}^{2^n-1} (-1)^{ik} p_k \right)^m \tag{3}$$

For the RHS of Lemma 1, using Lemma 2, we get

$$\begin{aligned} \boldsymbol{W}\boldsymbol{M}^m |0\rangle &= \boldsymbol{W}(\frac{1}{2^n}\boldsymbol{W}\boldsymbol{\Gamma}\boldsymbol{W})^m |0\rangle = \boldsymbol{W}(\frac{1}{2^n}\boldsymbol{W}\boldsymbol{\Gamma}^m\boldsymbol{W}) |0\rangle \\ &= \boldsymbol{\Gamma}^m\boldsymbol{W} |0\rangle = \boldsymbol{\Gamma}^m\vec{\boldsymbol{1}} \end{aligned} \tag{4}$$

Then

$$(WM^m |0\rangle)_i = \Gamma_{ii}^m = \left(\sum_{k=0}^{2^n-1} (-1)^{ik} p_k \right)^m = \lambda_i^m \tag{5}$$

Therefore,

$$\boldsymbol{\lambda}^m = \boldsymbol{W}\boldsymbol{M}^m |0\rangle \tag{6}$$

Lemma 2: For a given $\boldsymbol{p}$, $\boldsymbol{M}$ can be written as

$$\boldsymbol{M} = \frac{1}{2^n}\boldsymbol{W\Gamma W}$$

where $\boldsymbol{W}$ is Walsh-Hadamard Transform, and $\boldsymbol{\Gamma}$ is a diagonal matrix with $\Gamma_{ii} = \sum_{k=0}^{2^n-1}(-1)^{ik}p_k$.
Proof of Lemma 2:
By applying Singular Value Decompostion (SVD), $\boldsymbol{M}$ can be written as $\boldsymbol{M} = \boldsymbol{V\Gamma V}^{-1}$ where we assume that $\boldsymbol{V} = \boldsymbol{W}$ and $\boldsymbol{V}^{-1} = \frac{1}{2^n}\boldsymbol{W}$. The matrix $\boldsymbol{\Gamma}$ can then be written as

$$\boldsymbol{\Gamma} = \boldsymbol{V}^{-1}\boldsymbol{MV}$$

where $M_{ij} = p_{i\oplus j}$. We just have to prove that $\boldsymbol{\Gamma}$ is a diagonal matrix. By the definition of the Walsh-Hadamard Transform, $W_{ij} = (-1)^{ij}$, where ij is the bitwise inner product between two n-bit strings i and j, modulo 2. We have

$$\boldsymbol{\Gamma} = \frac{1}{2^n}\boldsymbol{WMW} = \frac{1}{2^n}\boldsymbol{RW} \tag{7}$$

where $\boldsymbol{R} = \boldsymbol{WM}$. Thus, R_{ij} can be written as

$$R_{ij} = \sum_{r=0}^{2^n-1} W_{ir}M_{rj} = \sum_{r=0}^{2^n-1}(-1)^{ir}p_{r\oplus j} \tag{8}$$

$$(RW)_{ij} = \sum_{s=0}^{2^n-1} R_{is}W_{sj} = \sum_{r=0}^{2^n-1}\sum_{s=0}^{2^n-1}(-1)^{ir\oplus js}p_{r\oplus s} = \sum_{k=0}^{2^n-1}(-1)^{jk}\left(\sum_{r=0}^{2^n-1}(-1)^{(i\oplus j)r}\right)p_k \tag{9}$$

After some simplifications,

$$\Gamma_{ij} = \frac{1}{2^n}(RW)_{ij} = \begin{cases} \sum_{k=0}^{2^n-1}(-1)^{ik}p_k, & i = j \\ 0, & \text{otherwise} \end{cases} \tag{10}$$

Thus, $\boldsymbol{\Gamma}$ is a diagonal matrix.

II. Jensen-Shannon Divergence (JSD)

The JSD is a method used to measure the similarity between two probability distributions $\boldsymbol{p}$ and $\boldsymbol{q}$. The JSD ranges between 0 and 1. The lower the JSD, the closer the two distributions are. The JSD between $\boldsymbol{p}$ and $\boldsymbol{q}$ is calculated as

$$JSD(\boldsymbol{p},\boldsymbol{q}) = \frac{1}{2}D(\boldsymbol{p}||\boldsymbol{m}) + \frac{1}{2}D(\boldsymbol{q}||\boldsymbol{m}) \tag{11}$$

where

$$\boldsymbol{m} = \frac{1}{2}(\boldsymbol{p}+\boldsymbol{q}) \tag{12}$$

$$D(\boldsymbol{P},\boldsymbol{Q}) = \sum_x \boldsymbol{P}(x)log(\frac{\boldsymbol{P}(x)}{\boldsymbol{Q}(x)}) \tag{13}$$

III. Constructing of the mitigation matrix

The following algorithm summarizes our proposed protocol for constructing the mitigation matrix Q_m for a new depth m not included in our *training set of depths.*

Algorithm 1 Proposed Protocol

Require: Choose a *training set* $\boldsymbol{T} = [1, m_{max}]$.

1: Choose circuit depth $m \in T$.

2: Sample a random sequence $s \in S_m$. The set of all length m sequences of one-qubit Clifford gates applied independently on each qubit, followed by an inverse gate for this sequence.

3: Obtain an estimate $\hat{\boldsymbol{q}}(m, s, |\boldsymbol{in}\rangle)$ of the probability distribution over the 2^n possible measurement outcomes.

4: Repeat steps 2-3 for K times to obtain an estimate of the average probability distribution $\hat{\boldsymbol{q}}(m, |\boldsymbol{in}\rangle)$ where

$$\hat{\boldsymbol{q}}(m, |\boldsymbol{in}\rangle) = \frac{1}{K} \sum \hat{\boldsymbol{q}}(m, s, |\boldsymbol{in}\rangle)$$

5: Apply an input-specific permutation matrix $\boldsymbol{\Pi}_{in}$ on $\hat{\boldsymbol{q}}(m, |\boldsymbol{in}\rangle)$ where

$$\Pi_{in_{ij}} = \begin{cases} 1 & if\, i \oplus j = in \\ 0 & otherwise \end{cases}$$

6: Apply Walsh-Hadamard transform $\boldsymbol{W}$ on $\hat{\boldsymbol{q}}(m, |\boldsymbol{in}\rangle)$ to obtain

$$\boldsymbol{\Lambda}(m) = \boldsymbol{W} \hat{\boldsymbol{q}}(m, |\boldsymbol{in}\rangle)$$

7: Repeat steps 1-6 for all $m \in T$.

8: For each parameter $\Lambda_i(m) \in \boldsymbol{\Lambda}(m)$, fit the model

$$\Lambda_i(m) = A_i \lambda_i^m$$

9: Apply an Inverse Walsh-Hadamard transform on $\boldsymbol{\lambda}$ to obtain the error rates of the Pauli Channel

$$\boldsymbol{p} = \boldsymbol{W}^{-1} \boldsymbol{\lambda}$$

10: Construct the input-specific spam error matrix $\boldsymbol{N}_{in} = \boldsymbol{W}^{-1} \boldsymbol{A} \boldsymbol{W}$ where $\boldsymbol{A}$ is a diagonal matrix with whose diagonal entries A_i.

11: Construct the input-specific average gate error matrix $\boldsymbol{M}_{in}$ where $M_{in_{ij}} = p_{i \oplus j}$ ($\oplus$ is the bitwise exclusive-Or operator).

12: Repeat Steps 1-11 for all $|\boldsymbol{in}\rangle \in \{|\boldsymbol{0}\rangle, |\boldsymbol{1}\rangle, \ldots, |\boldsymbol{2^n - 1}\rangle\}$.

13: For all basis input states, estimate $\hat{\boldsymbol{q}}(m', |\boldsymbol{in}\rangle)$ as

$$\boldsymbol{q}'(m', |\boldsymbol{in}\rangle) = \boldsymbol{N}_{in} \boldsymbol{M}_{in}^{m'} |\boldsymbol{in}\rangle$$

14: Construct the mitigation matrix $\boldsymbol{Q}_{m'}$ where

$$\boldsymbol{Q}_{m'} = [\boldsymbol{q}'(m', |\boldsymbol{0}\rangle), \boldsymbol{q}'(m', |\boldsymbol{1}\rangle), \ldots, \boldsymbol{q}'(m', |\boldsymbol{2^n - 1}\rangle)]$$

IV. Other Quantum Computers

In this section, we show the results of evaluating the accuracy of the model where we include the IBM Q Athens quantum computer. The Athens quantum computer is not included in the main manuscript since it is retired by IBM. Hence, we could not include the results of the mitigation. Similar to Figure 1 in the main manuscript, Figure 1 below presents the computed JSD for different quantum computers while varying m_{max} with the addition of IBM Q Athens that demonstrates JSD values that range between 0.005 and 0.08. Figure 2 presents the average and standard deviation for the test JSD values for the different quantum computers including IBM Q Athens that demonstrates an average test JSD between 0.02 and 0.04 for the different m_{max} values.

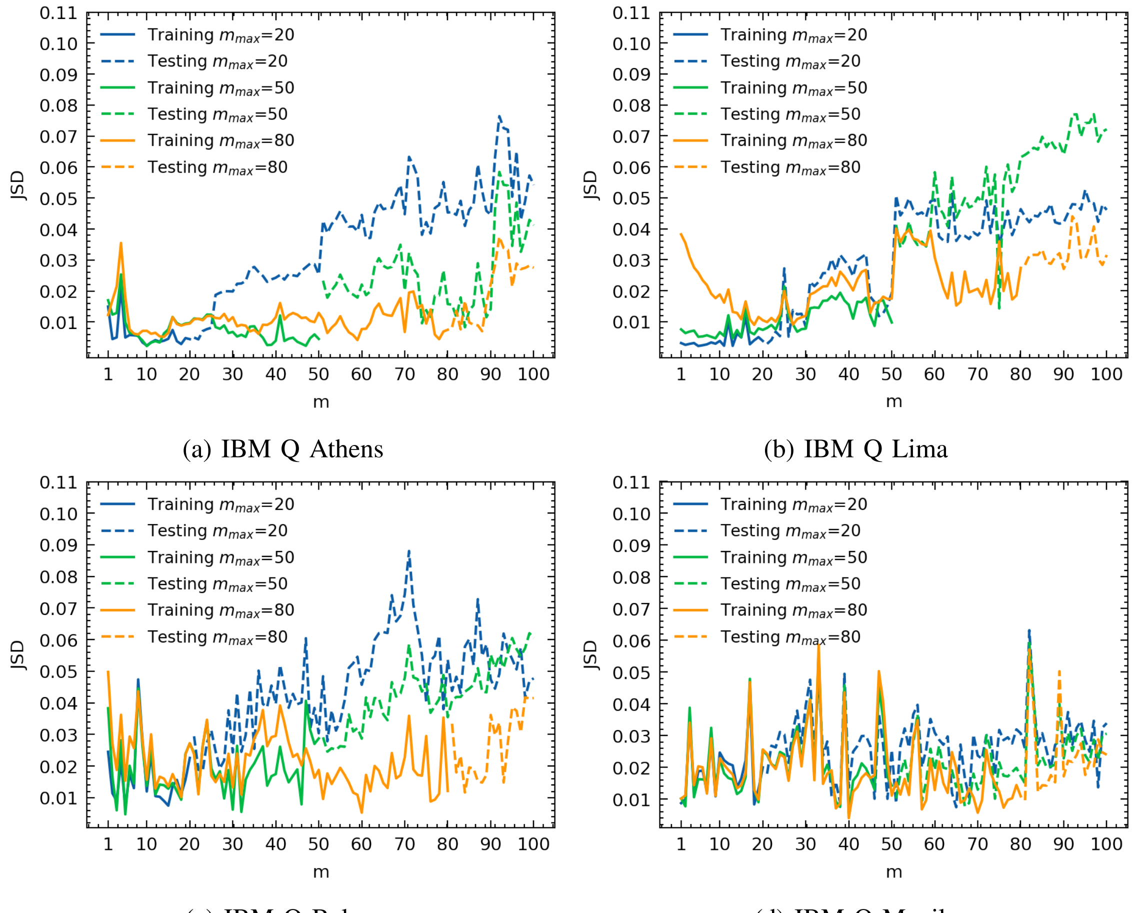

(a) IBM Q Athens

(b) IBM Q Lima

(c) IBM Q Belem

(d) IBM Q Manila

Fig. 1: $JSD(\hat{\boldsymbol{q}}(m, |\boldsymbol{in}\rangle), \boldsymbol{q}'(m, |\boldsymbol{in}\rangle))$ for training sets of depths T and testing sets of depths T' with variable maximum training depth $m_{max} \in \{20, 50, 80\}$ on different IBMQ 5-qubit quantum computers.

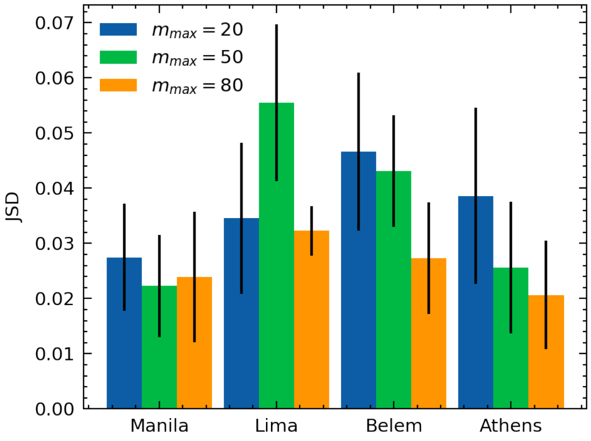

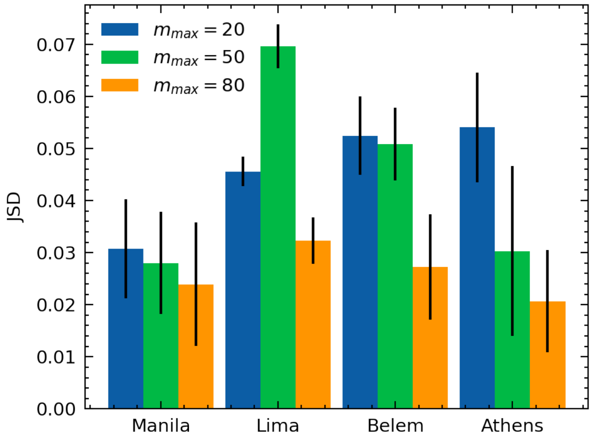

Fig. 2: The average and standard deviation of $JSD(\hat{\boldsymbol{q}}(m, |\mathbf{0}\rangle), \boldsymbol{q}'(m, |\mathbf{0}\rangle))$; (a) over all depths $m \in [m_{max} + 1, 100]$ and (b) over depths $m \in [80, 100]$ while varying the maximum training depth m_{max} on different IBM Q 5-qubit quantum computers.

V. Comparing the performance with $\boldsymbol{p}_{ave}$ vs $\boldsymbol{p}_{in}$

In this section, we evaluate the mitigation power of $\boldsymbol{Q}_m$ when relying on the input specific error rate vectors $\boldsymbol{p}(|\boldsymbol{in}\rangle)$ to construct $\boldsymbol{M}_{in}$ for all inputs compared to relying on the average error rate $\boldsymbol{p}_{avg} = \frac{1}{2^n}\sum_{in=0}^{2^n-1} \boldsymbol{p}(|\boldsymbol{in}\rangle)$ to construct $\boldsymbol{M}_{in} = \boldsymbol{M}_{avg}$. $\boldsymbol{N}_{in}$ remains input specific. Similar to Figure 3 in the main manuscript, Figure 3 below compares the average JSD between the ideal outputs and each of the unmitigated outputs, the mitigated outputs by the MEM protocol, and the mitigated outputs by $\boldsymbol{Q}_m$ using $\boldsymbol{p}_{ave}$ in addition to the mitigated outputs by $\boldsymbol{Q}_m$ using $\boldsymbol{p}(|\boldsymbol{in}\rangle)$. Figure 3 shows that the difference between using p_{ave} and $\boldsymbol{p}(|\boldsymbol{in}\rangle)$ for the construction of $\boldsymbol{Q}_m$ is negligible for the different quantum computers indicating consistent SPAM-free error rates for all inputs.

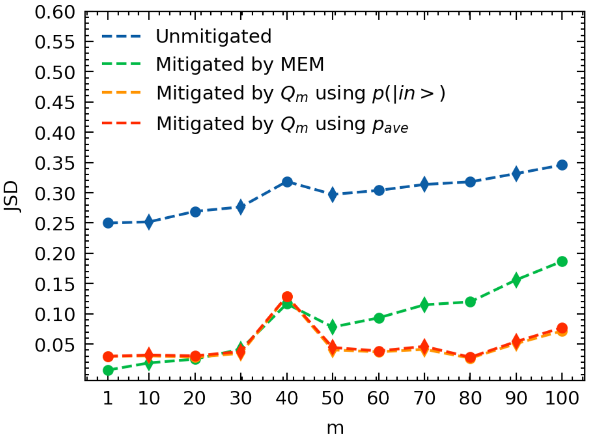

(a) IBM Q Lima

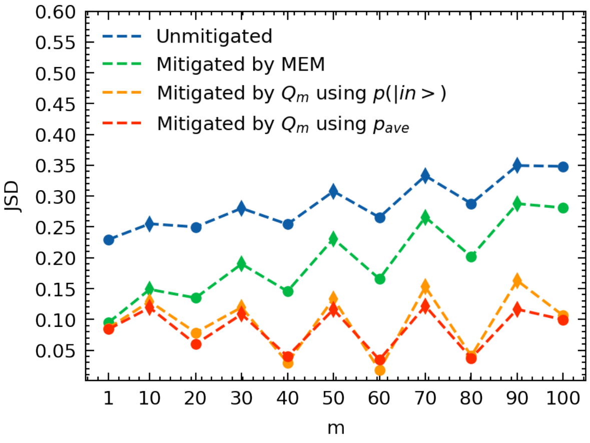

(b) IBM Q Manila

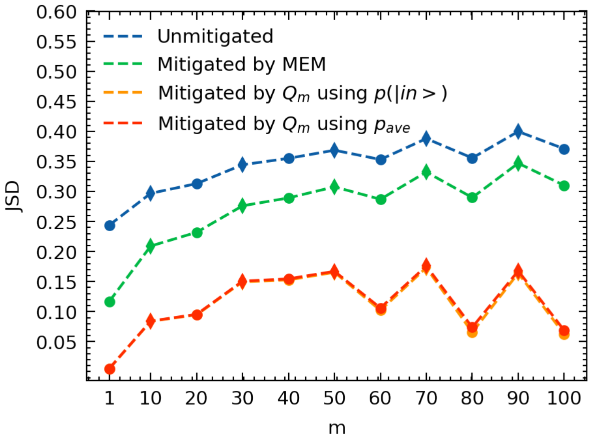

(c) IBM Q Belem

Fig. 3: Average JSD between the ideal output $|\boldsymbol{in}\rangle$ and each of the unmitigated outputs $\hat{\boldsymbol{q}}(m, s, |\boldsymbol{in}\rangle)$, mitigated outputs by the MEM protocol, and mitigated outputs by our proposed noise model using $\boldsymbol{p}(|\boldsymbol{in}\rangle)$ and using $\boldsymbol{p}_{ave}$ for each depth m on IBM Q 5-qubit quantum computers.

VI. Average JSD for Different Input States

In this section, we elaborate further on Figure 3 in the main manuscript and show the average JSD between the ideal output $|in\rangle$ and the mitigated output by our proposed protocol for each depth m and input state $|in\rangle$ (Figure 4). Figure 5 presents the average and standard deviation of JSD between the ideal output and mitigated output by our proposed protocol over all depths for each input state $|in\rangle$. We notice that, for the different quantum computers, some input states demonstrate lower JSD values compared to other input states. The Lima quantum computer presents the best average JSD in the worst input state with a minimum of 0.0023 and a maximum of 0.107 followed by the Belem quantum computer showing a minimum of 0.074 and a maximum of 0.202 and the Manila quantum computer showing a minimum of 0.031 and a maximum of 0.280. Table 1 shows the average reduction in the test JSD using the proposed protocol over the unmitigated data is about 58.33%, 61.04%, and 85.82% for the Belem, Manila, and Lima computers, respectively, compared to a reduction of 16.67%, 27.12%, and 72.77% for the Belem, Manila, and Lima computers, respectively, using the MEM protocol. We report upto 69% improvement and on average 40% improvement compared to the MEM protocol.

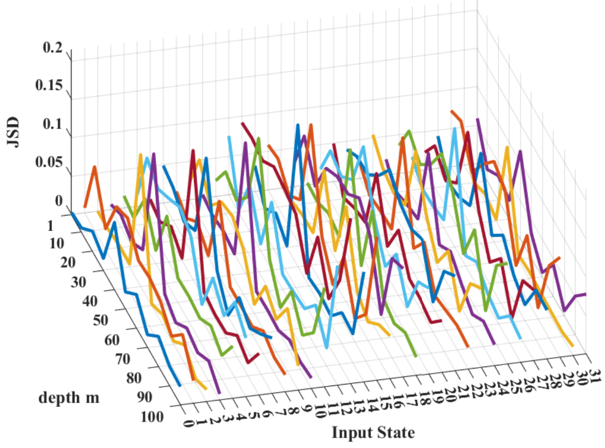

(a) IBM Q Lima

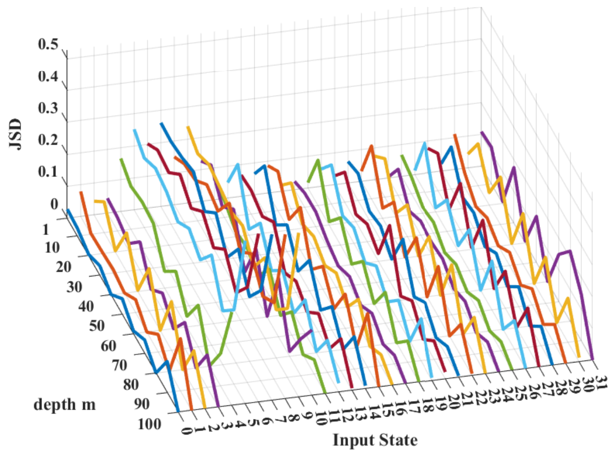

(b) IBM Q Manila

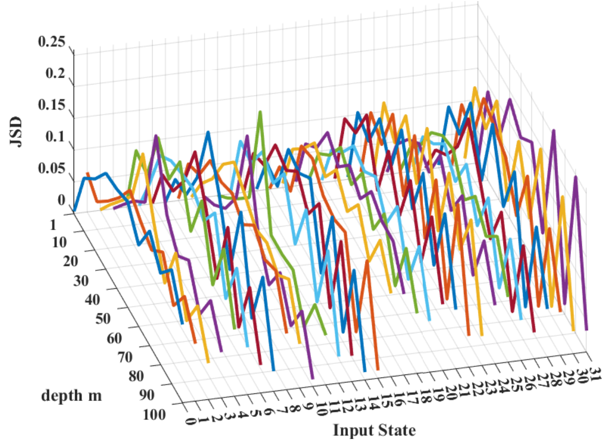

(c) IBM Q Belem

Fig. 4: Average JSD between the ideal output $|in\rangle$ and the mitigated outputs by our proposed noise model for each depth m and each input state $|in\rangle$ on IBM Q 5-qubit quantum computers.

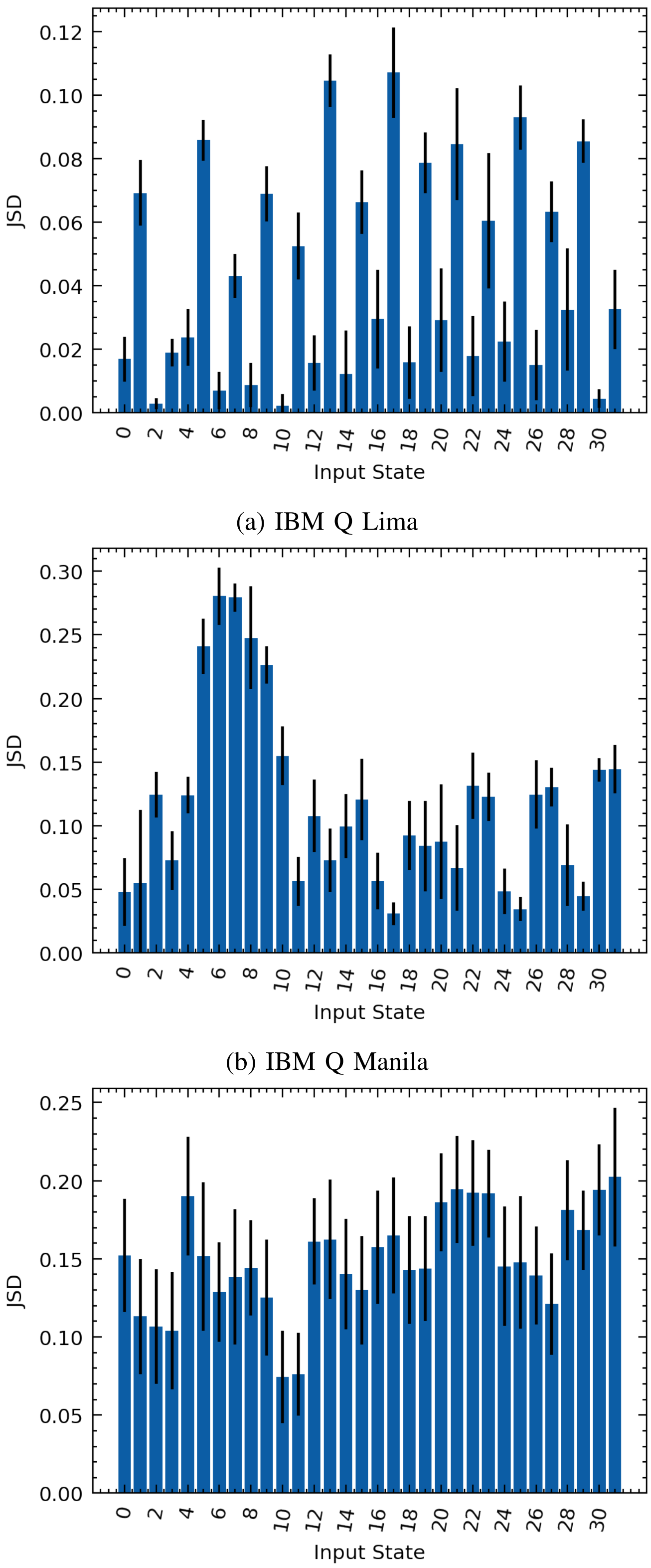

(a) IBM Q Lima

(b) IBM Q Manila

(c) IBM Q Belem

Fig. 5: Average and standard deviation of JSD over all depths m between the ideal output $|\boldsymbol{in}\rangle$ and the mitigated output by our proposed noise model for each input state $|\boldsymbol{in}\rangle$ on IBM Q 5-qubit quantum computers.

TABLE I: Average test JSD between the ideal output $|in\rangle$ and each of the unmitigated output, mitigated by the proposed protocol output, and mitigated by the MEM protocol output for the different quantum computers.

	m	Unmitigated JSD	MEM JSD	Mitigated by Proposed Protcol JSD	MEM improvement %	Proposed improvement %
Belem	10	0.30	0.21	0.08	30.00	73.33
	30	0.34	0.28	0.15	17.65	55.88
	50	0.37	0.31	0.17	16.22	54.05
	70	0.39	0.33	0.18	15.38	53.85
	90	0.40	0.35	0.17	12.50	57.50
	mean	0.36	0.30	0.15	16.67	58.33
Manila	10	0.26	0.15	0.12	42.31	53.85
	30	0.28	0.19	0.11	32.14	60.71
	50	0.31	0.23	0.12	25.81	61.29
	70	0.33	0.27	0.12	18.18	63.64
	90	0.35	0.29	0.12	17.14	65.71
	mean	0.31	0.23	0.12	27.12	61.04
Lima	10	0.25	0.02	0.03	92.00	88.00
	30	0.28	0.04	0.04	85.71	85.71
	50	0.30	0.08	0.04	73.33	86.67
	70	0.31	0.12	0.05	61.29	83.87
	90	0.33	0.16	0.05	51.52	84.85
	mean	0.29	0.08	0.04	72.77	85.82

VII. Comparison against RNZE

We compared the proposed technique against Richardson Zero Noise Extrapolation (RZNE) as requested. The following is the method we adopted to have as fair as comparison.

Richardson Zero Noise Extrapolation has two main components: noise scaling and then extrapolation. Global folding is a method for amplifying the noise by mapping a quantum circuit G as follows [1]:

$$G \to GG^{\dagger}G \tag{14}$$

This makes the circuit longer, which adds more noise, while keeping its effect unchanged ($G^{\dagger} = G^{-1}$). Folding a circuit is done using a scale factor λ, an integer which corresponds to how much the length of the circuit is scaled. The minimum scale factor is one which corresponds to $G \to G$ (i.e. no folding), $\lambda = 3$ corresponds to $G \to GG^{\dagger}G$, $\lambda = 5$ corresponds to $G \to GG^{\dagger}GG^{\dagger}G$, and so on.

For a given state ρ, we denote by $P(|0\rangle) = \langle 0|\rho|0\rangle$. For a given n-qubit circuit of depth m and input $|0\rangle$, we perform RZNE as follows [2]:

1) Repeat steps 2-3 for each $\lambda \in [1, 3, 5, 7, 9]$.
2) Scale the circuit by λ.
3) Measure $P(|0\rangle)_{unmitigated}$.
4) Fit the results to the model:

$$E_{Rich}(\lambda) = c_0 + c_1\lambda + ... + c_{n-1}\lambda^{n-1} \tag{15}$$

   where n is the given number of data points ($n = 6$ in this case).
5) Estimate $P(|0\rangle)_{mitigated}$ at $\lambda = 0$ i.e.

$$P(|0\rangle)_{mitigated} = E_{Rich}(0) \tag{16}$$

S_m is the set of all length m sequences of one-qubit Clifford gates applied independently on each qubit, followed by an inverse gate for this sequence. This given, we performed the Richardson ZNE as follows:

1) Repeat steps 2-4 for each depth $m \in [1, 10, 20, 30, 40, 50, 60, 70, 80, 90, 100]$

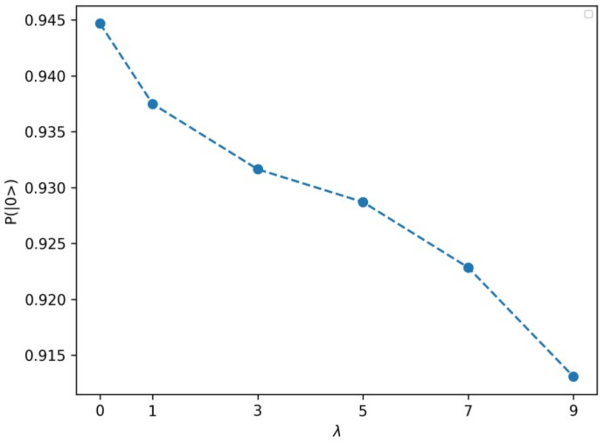

Fig. 6: RZNE performed on a circuit of depth $m = 100$ executed on Qiskit Aer simulator with with noise model of IBMQ's *"Lima"* five-qubit quantum computer. The true zero-noise value $P\,|\mathbf{0}\rangle = 1$.

2) Sample 100 random sequences of gates $s \in S_m$.
3) Perform RZNE on each circuit to obtain $P(m, s, |\mathbf{0}\rangle)_{mitigated}$
4) Obtain an estimate of the average $P(m, |\mathbf{0}\rangle)_{mitigated}$ by averaging over all sequences

We compare the RNZE results with our following experiment [3]:

1) Repeat steps 2-4 for each depth $m \in [1, 10, 20, 30, 40, 50, 60, 70, 80, 90, 100]$
2) Sample 100 random sequences of gates $s \in S_m$.
3) Obtain an estimate $\hat{\boldsymbol{q}}(m, s, |\mathbf{0}\rangle)$ of the probability distribution over the 2^n possible measurement outcomes.
4) Obtain an estimate of the noisy average probability distribution $\hat{\boldsymbol{q}}(m, |\mathbf{0}\rangle)$ by averaging over all sequences
5) Follow our paper approach to generate as $\boldsymbol{Q}_m = \boldsymbol{W}^{-1}\boldsymbol{A}\boldsymbol{W}\boldsymbol{M}^m$
6) Estimate the mitigated average probability distribution $\boldsymbol{q}(m)_{mitigated} = \boldsymbol{Q}_m^{-1}\hat{\boldsymbol{q}}(m\,|\mathbf{0}\rangle)$
7) Compute $P(m, |\mathbf{0}\rangle)_{mitigated} = \left\langle \mathbf{0}|\boldsymbol{q}(m)_{mitigated}|\mathbf{0}\right\rangle$

We generated our data by running 100 random circuits for each depth m with 1024 shots per circuit using IBMQ Aer simulator with different noise models for each of the IBMQ Lima and Belem quantum computers [3]. We used $\langle\mathbf{0}|\boldsymbol{\rho}|\mathbf{0}\rangle$ as our evaluation metric since it's used in [2] to evaluate RZNE.

While RZNE is circuit-dependent which means it can not be applied on any circuit of any depth without considering average noise, our protocol is generic since it can be used to mitigate the results of any random circuit of depth m. On the other side, RZNE is more computationally efficient since it does not need to compute the average noisy distributions over different circuit depths. It only requires replicating the circuit for different scaling factors. However, RZNE becomes relatively less efficient over large depths.

References

[1] "https://mitiq.readthedocs.io/en/v0.2.0/guide/guide-zne.html," Jan 2023.

[2] T. Giurgica-Tiron, Y. Hindy, R. LaRose, A. Mari, and W. J. Zeng, "Digital zero noise extrapolation for quantum error mitigation," in *2020 IEEE International Conference on Quantum Computing and Engineering (QCE)*. IEEE, 2020, pp. 306–316.

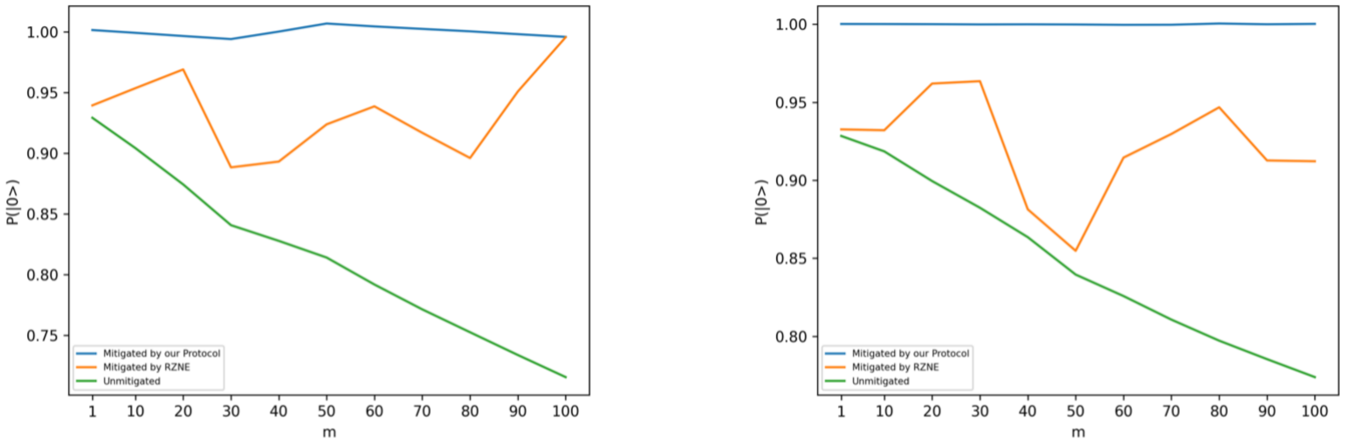

(a) (b)

Fig. 7: Comparison of mitigation methods averaged over 100 randomized benchmarking circuits executed on Qiskit Aer simulator with noise model of (a)IBM Lima quantum computer and (b) IBM Belem quantum computer for five-qubit quantum computer. The true zero-noise value $P\,|\mathbf{0}\rangle = 1$.

[3] A. Asfaw, A. Corcoles, L. Bello, Y. Ben-Haim, M. Bozzo-Rey, S. Bravyi, N. Bronn, L. Capelluto, A. C. Vazquez, J. Ceroni, R. Chen, A. Frisch, J. Gambetta, S. Garion, L. Gil, S. D. L. P. Gonzalez, F. Harkins, T. Imamichi, H. Kang, A. h. Karamlou, R. Loredo, D. McKay, A. Mezzacapo, Z. Minev, R. Movassagh, G. Nannicini, P. Nation, A. Phan, M. Pistoia, A. Rattew, J. Schaefer, J. Shabani, J. Smolin, J. Stenger, K. Temme, M. Tod, S. Wood, and J. Wootton. (2020) Learn quantum computation using qiskit. [Online]. Available: http://community.qiskit.org/textbook
